# High-flow nasal cannula (HFNC) vs continuous positive airway pressure (CPAP) vs nasal intermittent positive pressure ventilation as primary respiratory support in infants of ≥ 32 weeks gestational age (GA): study protocol for a three-arm multi-center randomized controlled trial

**DOI:** 10.1186/s13063-023-07665-7

**Published:** 2023-10-06

**Authors:** Rong Zhou, Tao Xiong, Jun Tang, Yi Huang, Wenli Liu, Jun Zhu, Chao Chen, Lingyue Gong, Ke Tian, Aoyu Wang, Dezhi Mu

**Affiliations:** 1 Department of Pediatrics, West China Second University Hospital, Sichuan University, No. 20, Section Three, South Renmin Road, Chengdu, China; 2 Key Laboratory of Birth Defects and Related Diseases of Women and Children (Sichuan University) Ministry of Education, Chengdu, China

**Keywords:** Neonates, Noninvasive ventilation, High-flow nasal cannula, Nasal intermittent positive pressure ventilation, Continuous positive airway pressure, Randomised controlled trial

## Abstract

**Background:**

Health problems in neonates with gestational age (GA) ≥ 32 weeks remain a major medical concern. Respiratory distress (RD) is one of the common reasons for admission of neonates with GA ≥ 32 weeks. Noninvasive ventilation (NIV) represents a crucial approach to treat RD, and currently, the most used NIV modes in neonatal intensive care unit include high-flow nasal cannula (HFNC), continuous positive airway pressure (CPAP), and nasal intermittent positive pressure ventilation. Although extensive evidence supports the use of NIPPV in neonates with a GA < 32 weeks, limited data exist regarding its effectiveness in neonates with GA ≥ 32 weeks. Therefore, the aim of this study is to compare the clinical efficacy of HFNC, CPAP, and NIPPV as primary NIV in neonates with GA ≥ 32 weeks who experience RD.

**Methods:**

This trial is designed as an assessor-blinded, three-arm, multi-center, parallel, randomized controlled trial, conducted in neonates ≥ 32 weeks’ GA requiring primary NIV in the first 24 h of life. The neonates will be randomly assigned to one of three groups: HFNC, CPAP or NIPPV group. The effectiveness, safety and comfort of NIV will be evaluated. The primary outcome is the occurrence of treatment failure within 72 h after enrollment. Secondary outcomes include death before discharge, surfactant treatment within 72 h after randomization, duration of both noninvasive and invasive mechanical ventilation, duration of oxygen therapy, bronchopulmonary dysplasia, time to achieve full enteral nutrition, necrotizing enterocolitis, duration of admission, cost of admission, air leak syndrome, nasal trauma, and comfort score.

**Discussion:**

Currently, there is a paucity of data regarding the utilization of NIPPV in neonates with GA ≥ 32 weeks. This study will provide clinical evidence for the development of respiratory treatment strategies in neonates at GA ≥ 32 weeks with RD, with the aim of minimizing the incidence of tracheal intubation and reducing the complications associated with NIV.

**Trial registration:**

Chinese Clinical Trial Registry: ChiCTR2300069192. Registered on March 9, 2023, https://www.chictr.org.cn/showproj.html?proj=171491.

## Administrative information

**Table:** 

Title {1}	High-flow nasal cannula (HFNC) vs continuous positive airway pressure (CPAP) vs nasal intermittent positive pressure ventilation as primary respiratory support in infants of ≥ 32 weeks gestational age (GA): study protocol for a three-arm multi-center randomized controlled trial
Trial registration {2a and 2b}	The trial has been registered at the Chinese Clinical Trial Registry: ChiCTR2300069192. Registered on March 9, 2023, https://www.chictr.org.cn/showproj.html?proj=171491. The register collects all items from the World Health Organization Trial Registration Data Set
Protocol version {3}	Version 4, Sep 15, 2023
Funding {4}	The study is funded by the National Key R&D Program
Author details {5a}	Rong Zhou, West China Second University Hospital, Sichuan University, Chengdu, China. 1490455039@qq.com Dr. Tao Xiong, West China Second University Hospital, Sichuan University; Key Laboratory of Birth Defects and Related Diseases of Women and Children, Ministry of Education, Chengdu, China. tao_xiong@126.com Dr. Jun Tang, West China Second University Hospital, Sichuan University; Key Laboratory of Birth Defects and Related Diseases of Women and Children, Ministry of Education, Chengdu, China. tj1234753@sina.com Yi Huang, West China Second University Hospital, Sichuan University, Chengdu, China. huangxiaoyixx@outlook.com Wenli Liu, West China Second University Hospital, Sichuan University, Chengdu, China. 1179016063@qq.com Jun Zhu, West China Second University Hospital, Sichuan University, Chengdu, China. 977343663@qq.com Chao Chen, West China Second University Hospital, Sichuan University, Chengdu, China. chaochenrt@163.com Lingyue Gong, RRT, RRT-NPS, West China Second University Hospital, Sichuan University, Chengdu, China. gonglingyue@outlook.com Ke Tian, West China Second University Hospital, Sichuan University, Chengdu, China. 740690908@qq.com Aoyu Wang, West China Second University Hospital, Sichuan University, Chengdu, China. 550118451@qq.com Dr. Dezhi Mu, West China Second University Hospital, Sichuan University; Key Laboratory of Birth Defects and Related Diseases of Women and Children, Ministry of Education, Chengdu, China. mudz@scu.edu.cn
Name and contact information for the trial sponsor {5b}	Dr. Tao Xiong Tel: 86 13730652940 Email: tao_xiong@126.com
Role of sponsor {5c}	The study funder is not involved in study design; collection, management, analysis, and interpretation of data; writing of the report; and the decision to submit the report for publication

## Introduction

### Background and rationale {6a}

Neonates with gestational age (GA) ≥ 32 weeks (including mid-term preterm infants (32–33^+5^ weeks), late preterm infants (34–36^+6^ weeks), and term infants (> 37 weeks)), constitute a significant proportion of births [1, 2]. Data collected from 107 countries revealed that moderate and late preterm infants, as well as term infants, accounted for 98.4% of all neonatal births [2]. The health concerns associated with these infants remain a major medical focus. Among these infants with GA ≥ 32 weeks, respiratory distress (RD) is a common cause for hospitalization [3–5]. RD is defined as the presence of at least two clinical symptoms for a minimum duration of 15 min, which may include tachypnea (respiratory rate > 60/min), subcostal and/or intercostal retractions, expiratory grunting, nasal flaring, and central cyanosis in room air [6]. A prospective study conducted in Switzerland reported that hospitalized neonates with GA ≥ 32 weeks accounted for 85.4% of all neonates with RD [7]. In the neonatal intensive care unit (NICU), RD led to hospitalization for 28.8% of late preterm infants and 15.6% of term infants [5].

Noninvasive ventilation (NIV) plays a crucial role in the treatment of RD, offering effective relief and reducing the occurrence of respiratory failure, tracheal intubation, and invasive mechanical ventilation (IMV) [8, 9]. Currently, the three most used NIV modes in the NICU are high-flow nasal cannula (HFNC), continuous positive airway pressure (CPAP), and nasal intermittent positive pressure ventilation [10–14]. CPAP, being the most widely employed NIV mode, has demonstrated its efficacy in enhancing oxygenation and increasing the success of extubation in earlier clinical trials [10–12, 15]. It is considered an effective choice for primary respiratory support for preterm infants, significantly reducing the need for IMV [16, 17]. HFNC, favored by nursing staff for its ease of use and ability to minimize nasal trauma, has gained extensive utilization in neonates [18–20]. While NIPPV, a relatively newer NIV mode, has been studied in very preterm infants, evidence regarding its effectiveness in neonates with GA ≥ 32 weeks remains limited.

Many studies have shown that NIPPV is superior to CPAP in reducing tracheal intubation and apnea in neonates with GA < 32 weeks [21–24]. When compared to CPAP, NIPPV exhibits significant reductions in respiratory failure, apnea, tracheal intubation, and extubation failure among preterm infants born before 37 weeks [25–27]. However, there is a relative scarcity of evidence regarding the comparison between NIPPV and CPAP specifically in neonates with GA ≥ 32 weeks. Further studies in this population are warranted to provide a clearer understanding of the effectiveness of NIPPV in comparison to CPAP.

There is limited evidence comparing the effectiveness of NIPPV and HFNC in neonates with GA ≥ 32 weeks. Two randomized controlled studies [28, 29] have been conducted, one including neonates with GA less than 35 weeks and the other including neonates with GA less than 34 weeks. These studies reported no significant difference between HFNC and NIPPV in preventing treatment failure. However, it is important to note that the sample sizes of these two studies were relatively small, suggesting the need for larger-scale research to further investigate and validate these findings.

Several studies have provided evidence supporting the superior outcomes of NIPPV over CPAP in terms of reduced treatment failure rates and tracheal intubation incidents [21–24]. However, due to the higher cost and greater technical complexity of NIPPV equipment, many clinical centers prefer to use CPAP as the primary respiratory support for neonates [30]. It has been reported that neonates with higher respiratory scores [31] have a higher increase in the need for respiratory support within 24 h [32]. In the case of neonates with GA ≥ 32 weeks, some individuals present with severe RD, and early implementation of more advanced NIV methods may potentially prevent the exacerbation of their condition, consequently reducing the incidence of tracheal intubation and mechanical ventilation. Therefore, our proposed research plan includes a subgroup analysis based on the severity of RD, aiming to investigate the effectiveness of NIPPV, CPAP, and HFNC in preventing treatment failure among neonates with varying degrees of RD severity.

Therefore, we will conduct a multicenter randomized controlled trial to investigate the effectiveness and safety of NIPPV in neonates with GA ≥ 32 weeks with RD and select the best primary NIV mode for these infants. We will compare the clinical effectiveness and safety in preventing treatment failure of HFNC, CPAP, and NIPPV as primary respiratory support in neonates with GA ≥ 32 weeks with RD through this study.

### Objectives {7}

The objective of this study is to compare the clinical effectiveness of HFNC, CPAP, and NIPPV as primary respiratory support in neonates with RD who are ≥ 32 weeks GA in reducing the need for IMV. We hypothesize that NIPPV is more effective than CPAP and HFNC in preventing treatment failure in neonates ≥ 32 weeks GA.

### Trial design {8}

This trial is designed as a three-arm, multicenter, parallel, randomized controlled trial with assessor blinding, with random allocation in a 1:1:1 ratio. It is a superiority clinical trial.

## Methods: participants, interventions, and outcomes

### Study setting {9}

This trial will be conducted at three centers: West China Second University Hospital, Sichuan University, Jinjiang Campus; West China Second University Hospital, Sichuan University, Ren Nan Campus; and Sichuan Provincial Children’s Hospital, which are tertiary hospitals equipped with ventilators capable of providing HFNC, CPAP, and NIPPV. Those participating centers have experienced medical staff who possess the necessary skills and expertise in treating and caring for infants receiving NIV. To ensure efficient data collection and management, the research data from each center will be transmitted and shared using electronic documents. This approach facilitates streamlined data exchange and minimizes the risk of data loss or errors during the research process. Standardized protocols for data collection, including relevant clinical parameters and outcomes, will be established to maintain consistency across all participating centers.

### Eligibility criteria {10}

#### Inclusion criteria

The inclusion criteria for infants participating in the trial are as follows:

1. They are born at ≥ 32 weeks GA; and
2. Their birth weight ≥ 1200 g, and
3. RD occurred within 24 h after birth (RD is defined as the presence of any two or more of the following symptoms: tachypnea, chest retraction, or grunting) [33].

#### Exclusion criteria

Infants will be excluded from the trial if they meet any of the following criteria:

1. They have a history of prior trachea intubation or require trachea intubation as determined by the attending pediatrician; or
2. They have major congenital anomalies or chromosomal abnormalities; or
3. They are suspected congenital lung diseases, malformations, or pulmonary hypoplasia; or
4. They have neuromuscular disorders; or
5. They have known air leak syndrome; or
6. Their parents don’t provide agreement or refuse to allow the infants before randomization.

### Who will take informed consent? {26a}

Guardian’s consent must be obtained before enrolling if the infant is eligible after admission. Full verbal and written informed consent must be included. The informed consent will be obtained by personnel with good clinical practice (GCP) qualifications.

### Additional consent provisions for collection and use of participant data and biological specimens {26b}

This study does not involve the collection of biological samples.

## Interventions

### Explanation for the choice of comparators {6b}

Given the established safety profiles and widespread usage of CPAP, NIPPV, and HFNC in neonatal care [16, 17, 25, 34], it is reasonable to consider these respiratory support modes as both control and intervention groups in this study. We plan to conduct paired comparisons; each group will serve as both a control and an intervention group.

### Intervention description {11a}

Infants enrolled in the study will be randomly assigned to one of the three groups: the HFNC group, CPAP group, or NIPPV group. Once assigned to a specific group, the infants will immediately begin the corresponding intervention based on the assigned group.

Before applying the assigned intervention, each newborn will be assessed the severity of RD using the Silverman Andersen score (SAS) [31]. This approach ensures a standardized and systematic evaluation of RD severity before commencing the assigned intervention, allowing for a consistent baseline comparison across the different treatment groups. Then, the assigned NIV can be applied.

#### Ventilators

HFNC: HFNC will be provided by devices that deliver a blend of heated and humidified gas mixture of air and oxygen at gas flows exceeding 1 L/ min via binasal cannula, including but not limited to Drager VN300, Drager C500, Fabian, Mindray NB350.

CPAP: CPAP will be provided by devices with pressure that is measurable and controllable that transport heated and humidified gas via binasal nasal prongs or nasal masks, including but not limited to Drager VN300, Drager C500, Fabian, Mindray NB350.

NIPPV: NIPPV will be provided by devices that able to provide sufficient pressure mentioned in the protocol (as indicated below), including but not limited to Drager VN300, Drager C500, Fabian, Mindray NB350. “NIPPV” in this trial is a broad term, a form of noninvasive respiratory support combining CPAP with intermittent higher pressure, including traditional NIPPV and Bi-level positive airway pressure (BiPAP) [35, 36]. When the pressure of BiPAP cannot meet the pressure required by the protocol, it can be replaced with other ventilators that can provide sufficient pressure.

#### Interface

In the CPAP group and the NIPPV group, neonates will be provided with soft, short binasal nasal prongs or nasal masks that are specifically matched with the ventilator being used. The size of nasal prongs will be chosen based on the diameter of the nares, ensuring the largest size that fits comfortably without compressing the surrounding tissues, in accordance with the manufacturer’s recommendations. Similarly, the size of the nasal masks will be selected according to the manufacturer’s guidelines to ensure an appropriate fit and effective respiratory support. To minimize the risk of nasal trauma, nasal prongs and masks can be used alternately, depending on the clinical situation.

For the HFNC group, neonates will use binasal cannulas with a diameter smaller than 50% of the nares’ size. This selection aims to reduce the risk of excessive air pressure and ensures safe and effective delivery of the heated and humidified gas provided by the HFNC system [37].

#### Ventilatory management

In the HFNC group, the initial gas flow setting will be 6 L/min for all infants. The fraction of inspired oxygen (FiO_2_) will be adjusted to maintain pulse oximetry oxygen saturation (SpO_2_) between 90–94%, and the maximum FiO_2_ will be increased up to 0.40 to ensure adequate oxygenation. According to the SpO_2_ and arterial partial pressure of carbon dioxide (PaCO_2_) levels, the gas flow can be increased up to a maximum of 8 L/min.

In the CPAP group, the initial CPAP setting will be 6 cmH_2_O for all infants. FiO_2_ will be adjusted to maintain SpO_2_ between 90–94%. If necessary, the maximum FiO_2_ will be increased up to 0.40 to ensure adequate oxygenation. According to the SpO_2_ and PaCO_2_ levels, the CPAP can be increased up to a maximum of 8 cmH_2_O to optimize respiratory support and maintain appropriate oxygenation and ventilation.

In the NIPPV group, the starting parameters of ventilator will be set as follows:

- ❿ Positive end-expiratory pressure of 6 cmH_2_O (can be increased up to 8 cmH_2_O, according to the SpO_2_ levels);
- ❿ Peak inspiratory pressure (PIP) of 10–15 cmH_2_O (can be increased up to 25 cmH_2_O, according to the SpO_2_ and PaCO_2_ levels);
- ❿ Inspiratory time (IT) of 0.5 s (can be adjusted ranging from 0.4–0.6 s), and rate will be 30 breaths per minute (bpm) (can be increased up to 60 bpm, according to PaCO_2_ levels);
- ❿ FiO_2_ will be adjusted to maintain SpO_2_ between 90–94%, and the maximum FiO_2_ is 0.40.

#### Weaning from study interventions

The assigned intervention will be weaned based on the clinical assessment of infants and the following protocol:

- In HFNC group, the gas flow for infants can be decreased down to 2 L/min;
- In CPAP group, the setting pressure for infants can be decreased down to 4 cmH_2_O;
- In NIPPV group, the PIP and PEEP for infants can be decreased down to 8 cmH2O and 4 cmH_2_O respectively. The rate can be decreased down to 20 bpm.

The study intervention will be stopped when the parameters are reduced to the above and can maintain for at least 24 h with the following:

1. FiO_2_ ≤ 0.25;
2. SAS < 3;
3. No apnea that cannot be self-recovered.

If infants need FiO_2_ > 0.25 to maintain SpO_2_ at 90–94%, SAS > 3 points, or have apnea ≥ 1 time a day that cannot be self-recovered, the assigned intervention will be continued and reassessed after at least 24 h. After weaning of the intervention, neonates can receive continuous hood oxygen therapy, or low-flow oxygen therapy via nasal cannula, if needed. If reintroduction of NIV is required within 48 h after weaning, the previously assigned intervention will be used.

Pulmonary surfactant (Curosurf®) will be administered to infants with FiO_2_ > 0.30 and with RD syndrome (RDS) on chest x-ray or ultrasound [33]. A dose of 200 mg/kg will be administered by INSURE (intubation-surfactant-extubation) method. If the infant’s RD persists and FiO_2_ remains > 0.30 for 6–12 h after the first dose, an additional dose of 100 mg/kg will be given. PaCO_2_ will be monitored by blood gas analysis [38] or transcutaneous monitoring [39] according to local policy. SpO2, electrocardiogram (ECG), heart rate will be continuously monitored by bedside monitors. To relieve abdominal distension, each patient will be placed with an orogastric tube, through which gas can be aspirated according to nurses’ assessment.

### Criteria for discontinuing or modifying allocated interventions {11b}

The assigned interventions may be terminated or modified under the following circumstances:

1. Treatment failure criteria: If an infant meet any of the predefined treatment failure criteria, the assigned intervention will be terminated. These criteria may include specific clinical parameters or worsening of the infant’s condition that indicate the need for a different level of respiratory support or intervention;
2. Guardian request: If the guardian of an enrolled infant requests the termination of the assigned intervention, their decision will be respected, and appropriate actions will be taken based on the infant’s clinical condition;
3. Principal investigator’s decision: The principal investigator, who is responsible for the overall conduct of the study, may deem it necessary to terminate or modify the assigned intervention. This could be due to safety concerns for the subjects or other relevant factors that warrant a change in the treatment approach;
4. Ethics committee determination: If the ethics committee overseeing the study determines that there are significant safety issues or other ethical concerns that warrant the termination of the trial, they may recommend discontinuing the assigned interventions.

### Strategies to improve adherence to interventions {11c}

Before commencing the study, the principal investigator and team members will convene a meeting to review the study protocol, data collection and management procedures, and specific intervention details.

After discussion, we have decided to make the following modifications. Due to not all relevant healthcare personnel participating in the study being proficient in all modes of respiratory support, and the management and operation of respiratory support being crucial for the success of this trial, it was decided through discussion that training is necessary. The protocol manuscript has been distributed to the relevant personnel at the participating centers 45 days prior to recruitment. The principal investigator has conducted online meetings to explain the study protocol to all researchers, with a focus on intervention details. A dedicated discussion group has been established on a social media platform to facilitate prompt answering of any questions. Respiratory therapists who are skilled and knowledgeable about the protocol and principles have been assigned to each center to provide practical training on operational procedures and address related queries. Two weeks prior to the commencement of recruitment, all personnel involved in respiratory support operations and management were proficient in performing the relevant procedures according to the protocol.

To ensure the smooth implementation of the protocol as per requirements, the principal investigator will regularly supervise and assess the performance of healthcare personnel. Regular online summary meetings will be conducted to review any issues and challenges encountered during the trial period and to identify ways for improvement and solutions.

### Relevant concomitant care permitted or prohibited during the trial {11d}

All neonates including in the trial will receive standard supportive care such as blood tests, X-rays, antibiotics use, parenteral and enteral nutrition according to local policy.

### Provisions for post-trial care {30}

Not applicable. The interventions involved in this trial have minimal harm, and after the trial, the subjects will receive standard care according to local policies.

### Outcomes {12}

#### Primary outcome

The primary outcome is treatment failure within 72 h after randomization [34]. Treatment failure is defined as meeting one or more of the following criteria when the neonate has received maximal therapy for the assigned treatment:

1. Sustained increase in oxygen requirement: FiO_2_ > 0.40 to maintain SpO_2_ of 90%-94% for more than one hour.
2. Respiratory acidosis: potential of Hydrogen (pH) ≤ 7.20 and PaCO_2_ > 60 mm Hg.
3. Frequent or severe apnea despite drug therapy or respiratory support: two or more apnea events requiring bag-mask ventilation within 24 h, or three or more apnea events requiring intervention (stimulation, increasing oxygen or ventilation pressure) within one hour.
4. Emergency situations such as pulmonary hemorrhage, pneumothorax, heart failure, shock, etc., require endotracheal intubation, or it may be deemed necessary by the pediatrician.

#### Secondary outcomes

1. Death before discharge.
2. Surfactant treatment within 72 h after randomization.
3. Duration of both NIV and IMV.
4. Duration of oxygen therapy.
5. Bronchopulmonary dysplasia (BPD).
6. Time to achieve full enteral nutrition.
7. Necrotizing enterocolitis.
8. Duration of admission.
9. Cost of admission.
10. Air leak syndrome (including pneumothorax, pneumomediastinum, and pneumopericardium).
11. Nasal trauma, which will be assessed based on a clinical score [40].
12. Comfort score (Neonatal Pain, Agitation and Sedation Scale, N-PASS) [41].

### Participant timeline {13}

**Fig. 1:**
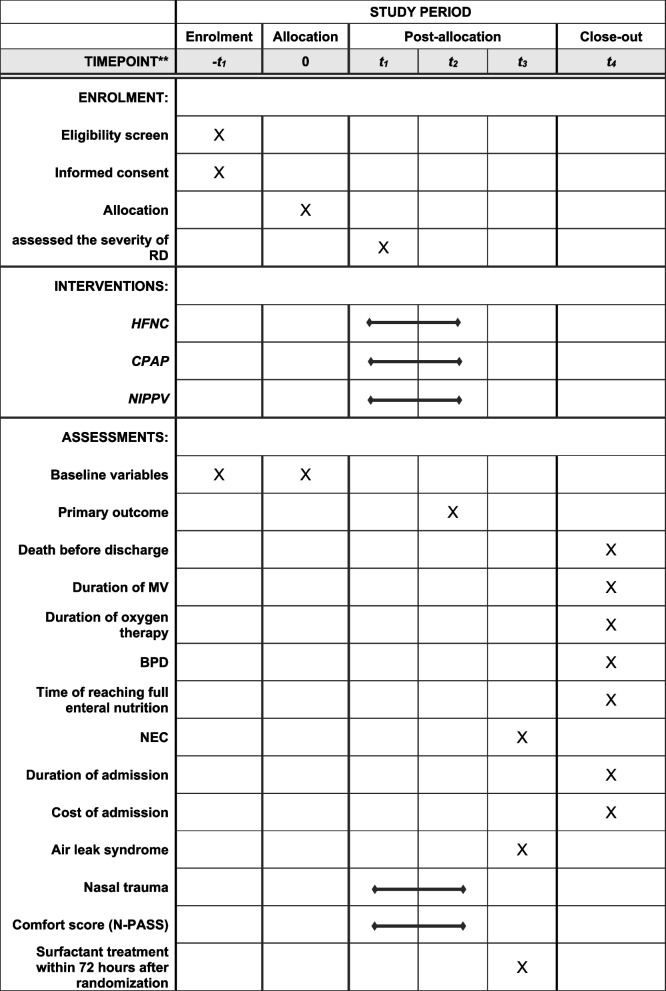
The schedule of recruitment, interventions, and assessments. t_1:_ immediately after randomisation; t_2:_ 72 h after randomisation; t_3:_ after weaning or failure of allocated treatment; t_4:_ first hospital discharge; RD: respiratory distress; HFNC: high-flow nasal cannula; CPAP: continuous positive airway pressure; NIPPV: nasal intermittent positive pressure ventilation; MV: mechanical ventilation; BPD: bronchopulmonary dysplasia; NEC: necrotizing enterocolitis; N-PASS: Neonatal Pain, Agitation and Sedation Scale

Figure 1 The schedule of recruitment, interventions, and assessments (See attachment 1).

### Sample size {14}

As this study aims to compare the effectiveness and safety of NIPPV, CPAP, and HFNC in infants with GA ≥ 32 weeks, we plan to conduct paired comparisons. Since no studies with identical content to this study have been conducted in the past, we will refer to the following studies for sample size calculation. A trial comparing CPAP and BiPAP including neonates of GA ≥ 34 weeks with transient tachypnea of the newborn, showed that BiPAP had lower treatment failure (CPAP: 20%, BiPAP: 7.9%, P: 0.03) [42]. In addition, a prospective randomised controlled trial comparing CPAP and HFNC in infants with GA ≥ 31 weeks showed higher treatment failure in the HFNC group (HFNC: 20.5%, CPAP: 10.2%, CI: 10.3) [34]. Therefore, we can calculate the sample size of this trial based on the data from the study [34], in which we will use the treatment failure of HFNC and CPAP as primary respiratory support in neonates as a reference. Considering the trial involving multiple comparisons, using an alpha error rate of 0.0167 and a power of 0.8, at least 252 infants should be enrolled in each arm calculated by PASS15.0.5 software, so the total sample size is 756. Considering 5% loss, the sample size would be no less than 795.

### Recruitment {15}

Each participating center’s NICU has established a dedicated position responsible for coordinating the admission and discharge of newborns. We will provide training to the personnel in this position. If a newborn with a gestational age of ≥ 32 weeks is admitted, they will immediately notify the recruitment personnel in charge of this study. All neonates who meet the eligibility criteria can be considered for inclusion in the study and will be recruited by personnel with GCP qualifications. Based on data from participating centers, we estimate that the recruitment period will span approximately 2 years to ensure an adequate sample size for the study.

## Assignment of interventions: allocation

### Sequence generation {16a}

Randomization stratification will be made by GA (< 37 and ≥ 37 weeks’) and by study centers. A 1: 1: 1 allocation ratio and variable block sizes will be used for random allocations within each stratum. Multiple births meeting eligibility criteria will be randomized individually.

### Concealment mechanism {16b}

The allocation of the randomization sequence is conducted through a confidential centralized telephone system, which is concealed from investigators at each of the study centers.

### Implementation {16c}

**Fig. 2:**
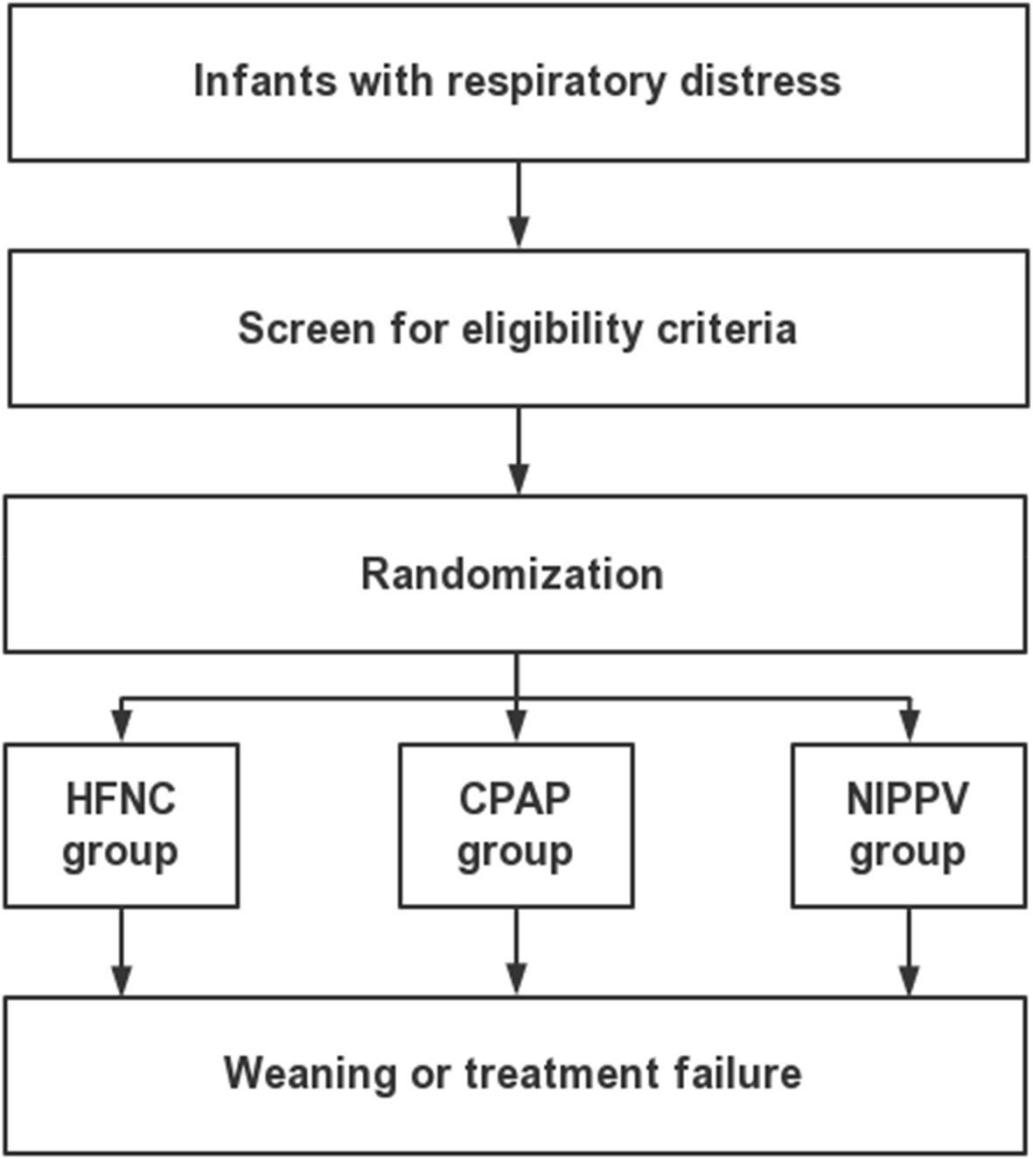
Flow diagram of the study

The randomization sequence is computer generated by dedicated personnel who are not involved in subject inclusion and intervention assignments. All neonates who meet the eligibility criteria can be considered for inclusion in the study and will be recruited by doctors involved in this trial. When an infant meets the eligibility criteria and guardian’s written consent has been obtained, the corresponding assignment based on the randomization sequence will be immediately applied to the infant by respiratory therapists involved in this trial (Fig. 2).

## Assignment of interventions: blinding

### Who will be blinded {17a}

In this trial, blinding of the healthcare professionals involved in delivering the respiratory support methods (doctors, nurses, and respiratory therapists) is not feasible due to the nature of the intervention; it is meaningless to blind the infants. To minimize risk of bias, we developed objective criteria for the primary outcome. Outcome assessors and data analysts will be blinded to the treatment allocation and not involved in the treatment process.

### Procedure for unblinding if needed {17b}

The design is open label with only outcome assessors and data analysts being blinded so unblinding will not occur.

## Data collection and management

### Plans for assessment and collection of outcomes {18a}

Prior to the commencement of the trial, the principal investigators will provide training to the data assessors to ensure their thorough understanding of the study protocol and the data to be collected. Clinical information will be collected at the following time points:

- Before randomization:
  Information on eligibility; baseline characteristics of study centers, infants, and mothers; diagnosis of respiratory system diseases.
- After randomization and before intervention:
  Assessment of the severity of RD using SAS.
- Following intervention:
  Ventilatory parameters, blood gas values: PaO_2_, PaCO_2_, pH; SpO_2_.
- Follow-up:
  Treatment failure within 72 h after randomization, surfactant treatment within 72 h after randomization, duration of both NIV and IMV, duration of oxygen therapy, BPD, time to reaching full enteral nutrition, necrotizing enterocolitis, cost of admission, duration of admission, and air leak syndrome (including pneumothorax, pneumomediastinum, and pneumopericardium), nasal trauma, comfort score.

The clinical scores involved in this study include the SAS [31], assessment of Nasal trauma based on a clinical score [40], and the Comfort score (N-PASS) [41]. The SAS can be used to assess the severity of respiratory distress in newborns and is an internationally recognized scoring tool [31, 33, 43]. To assess the severity of nasal trauma, we utilized a clinical scoring system developed by Fischer et al. [40]. This scoring system has been employed in several clinical studies for evaluating nasal trauma [44–46]. The score categorizes and rates nasal trauma in newborns and please refer to the reference for a detailed description [40]. In this study, we employed the N-PASS to assess the comfort of newborns undergoing non-invasive ventilation [41]. Multiple system reviews have demonstrated that N-PASS exhibits good measurement performance when used for acute and prolonged pain assessment in both full-term and preterm infants, as well as in specific clinical scenarios such as postoperative and mechanical ventilation situations [47–50].

### Plans to promote participant retention and complete follow-up {18b}

To promote participant retention, this study will provide detailed research information to guardians, including the study background, objective, intervention plan, and participant rights. This ensures that guardians understand the safety and importance of the study and are aware of their right to withdraw or terminate their participation at any time without prejudice. A good communication channel will be established with the guardians to facilitate their interaction with the research team and address any questions or concerns, providing them with reasonable support and assistance.

If the guardians request to no longer receive the originally assigned intervention, the reasons for withdrawal and time point of withdrawal will be fully documented, and researchers will seek consent from the guardians to collect relevant data. If the guardian consents, data collection prior to the withdrawal point will be conducted in accordance with the schedule of recruitment, interventions, and assessments (Fig. 1 in attachment 1). If the guardian refuses, the infants will be withdrawn from the study and replaced.

### Data management {19}

An electronic medical record form will be generated for each enrolled infant to facilitate data collection and management. Respiratory therapists and doctors involved will fill out bedside record forms, which includes ventilatory parameters, blood gas values (PaO_2_, PaCO_2_, pH), and SpO_2_. Data obtained from the bedside record forms and the hospital medical record system (HIS system) will be entered into the electronic medical record form by the data managers. These personnel will receive relevant training to ensure the accuracy, completeness, and confidentiality of the data. The electronic medical record form will be archived in real-time on the online system, with access restricted to authorized personnel only. The principal investigator will periodically review the data, identify missing or erroneous data, and urge local researchers to complete the necessary information (Fig. 2).

### Confidentiality {27}

Each enrolled patient has an independent number and center number, which can only be identified by combining the two. No researcher may disclose patient identity information outside the hospital.

### Plans for collection, laboratory evaluation and storage of biological specimens for genetic or molecular analysis in this trial/future use {33}

There is no plan to collect any biological material samples in this study.

## Statistical methods

### Statistical methods for primary and secondary outcomes {20a}

Statistical analysis will be performed on the basis of intention-to-treat analysis, according to the Consort reporting guidelines. The normality of continuous outcomes will be tested by Kolmogorov–Smirnov test. Mean difference with 95% confidence interval (CI) will be calculated for normally distributed outcomes and median difference with P25-P75 for non-normally distributed continuous outcomes. Continuous outcomes will be compared by the appropriate parametric (one- way ANOVA) or non-parametric (Mann–Whitney U) test, followed by post hoc test if needed. Dichotomous outcomes will be compared by Chi-square test. Multivariate regression analyses will also be conducted for selected outcomes, if required, and in that case, logistic regression, linear regression, or Cox regression analyses will be performed based on the type and distribution of variables. Specifically, if a baseline characteristic of the enrolled population shows a significant difference (*p* < 0.2) between the two groups in the univariate analysis, the results will be adjusted for that variable. To control for the error of multiple comparisons, the *P*-value of multiple comparisons will be corrected by Bonferroni method. All tests will be two-tailed, and *P* < 0.0167 will be considered significant.

### Interim analyses {21b}

No interim analysis will be conducted in this trial.

### Methods for additional analyses (e.g., subgroup analyses) {20b}

Subgroup analyses of the primary outcomes will be performed based on SAS (4–6 points, 7–10 points) [43] and GA (32W ≤ GA < 34W, 34W ≤ GA < 37W, GA ≥ 37W).

### Methods in analysis to handle protocol non-adherence and any statistical methods to handle missing data {20c}

For infants lost to follow-up, multiple imputation will be used to replace missing values for primary and secondary outcomes. The *P* < 0.0167 will be considered statistically significant.

### Plans to give access to the full protocol, participant level-data and statistical code {31c}

After the completion of the study and publication of relevant manuscripts, requests to obtain the related research data can be made by contacting the study principal investigator.

## Oversight and monitoring

### Composition of the coordinating centre and trial steering committee {5d}

The coordinating centre consists of the principal investigator, project coordinator, other researchers (neonatologists, nurses, respiratory therapists), and data analysts. The principal investigator is the overall responsible person for this study. The research team members will be responsible for the daily operation and running of the trial. The principal investigator and team members will hold online or necessary offline meetings periodically to ensure the smooth running of the trial.

The trial steering committee consists of the principal investigator, an independent neonatology specialist not involved in the trial, a data manager, and an ethics expert. The trial steering committee will be responsible for overseeing the overall progress of the trial, including trial design, ethical issues, data security, etc., and providing advice and decision-making support.

There is no any Patient and Public involvement.

### Composition of the data monitoring committee, its role and reporting structure {21a}

Even though the risk of this study is relatively low, we have established a Data Monitoring Committee (DMC) to enhance the quality and reliability of the research. The DMC consists of three pediatricians who are not involved in the study, along with a data manager and a statistician. They will regularly review the study data to evaluate its safety, implementation, and data quality. The DMC operates independently from the sponsors and has no conflicts of interest.

### Adverse event reporting and harms {22}

An adverse event (AE) refers to any unfavorable medical occurrence in a subject who has received an intervention, which may manifest as symptoms, signs, diseases, or laboratory abnormalities but does not necessarily have a causal relationship with the study intervention. Researchers will record all AEs truthfully, whether they are related to the study intervention. The AE records include the time, severity, duration, relationship with the intervention, measures taken, and outcome of the AE. When subjects experience AEs, researchers should take appropriate medical measures as necessary. All AEs should be followed up until they return to normal, stable, or baseline levels. A serious adverse event (SAE) is defined as an event that occurs during the clinical trial process and requires prolonged hospitalization, life-threatening or fatal outcomes, or causes severe or permanent disability. If any SAEs occur in the clinical study, the monitoring committee should be immediately notified, and a SAE form should be completed. If the committee determines that the SAE is related to the intervention, suspected and unexpected serious adverse reactions should be reported promptly to relevant departments, including all participating researchers and clinical trial institutions, ethics committees, and health authorities.

### Frequency and plans for auditing trial conduct {23}

The independent committee will conduct a review quarterly and as needed based on any reported AEs. Safety monitoring will begin from the start of trial recruitment. The committee will provide a report for each review, detailing all AEs and any protocol deviations.

### Plans for communicating important protocol amendments to relevant parties (e.g., trial participants, ethical committees) {25}

Any revisions that affect the rights of subjects and evaluation of trial results will be notified to the medical ethics committee and update the trial register. Other revisions that are confirmed by the principal investigator to be permissible will not be notified to the medical ethics committee but will be recorded. All revisions will be communicated to local investigators via emails.

### Dissemination plans {31a}

The results of this trial will be disseminated through conferences and peer-reviewed research publications both domestically and internationally. All peer-reviewed manuscripts generated by this trial will be submitted to the PubMed Central digital archive.

## Discussion

Innovation of this study: Currently, evidence for the use of NIPPV in neonates with GA ≥ 32 weeks is still insufficient. This study will supplement the data on the application of NIPPV in neonates with GA ≥ 32 weeks and compare the effectiveness and safety of the three most used modes in the NICU: CPAP, NIPPV, and HFNC. This will provide more evidence for clinical application of NIV in this population.

Limitations of this study: 1) Blinding of the healthcare professionals involved in the diagnosis and treatment of the neonates is not feasible due to the nature of the ventilation mode being used in the study. However, to minimize potential bias, objective primary outcome criteria have been established. These criteria will be used to assess the outcomes in a standardized and unbiased manner, reducing the impact of lack of blinding on the study results. 2) In this trial, the term “NIPPV” includes both traditional NIPPV and BiPAP. BiPAP is commonly categorized as a form of NIPPV [35]. Previous studies have indicated that both traditional NIPPV and BiPAP show comparable efficacy in preventing treatment failure and the need for MV [51]. Therefore, traditional NIPPV and BiPAP can be considered interchangeable in terms of their effectiveness. Considering that the maximum pressure of BiPAP is lower, traditional NIPPV can be used when the required pressure exceeds the upper limit of BiPAP.

Our study aims to generate robust clinical evidence regarding the efficacy and safety of noninvasive respiratory support in neonates with GA ≥ 32 weeks, and strive to minimize tracheal intubation and mechanical ventilation, and reduce complications related to NIV.

## Trial status

Protocol version: Version 4, Sep 15, 2023.

Recruitment start date: September 1, 2023.

Recruitment completion date: August 31, 2025 (estimated).

## Data Availability

This manuscript does not contain any data obtained during the study period. The datasets generated during the current study will be available by contacting the corresponding author upon reasonable request.

## Abbreviations

AE: Adverse event
BiPAP: Bi-level positive airway pressure
BPD: Bronchopulmonary dysplasia
bpm: Breaths per minute
CI: Confidence interval
CPAP: Continuous positive airway pressure
DMC: Data monitoring committee
ECG: Electrocardiogram
FiO_2_: Fraction of inspired oxygen
GA: Gestational age
GCP: Good clinical practice
HFNC: High-flow nasal cannula
INSURE: Intubation-surfactant extubation
IT: Inspiratory time
NICU: Neonatal intensive care unit
NIPPV: Nasal intermittent positive pressure ventilation
NIV: Noninvasive ventilation
IMV: Invasive mechanical ventilation
N-PASS: Neonatal Pain, Agitation and Sedation Scale
PaCO_2_: Arterial partial pressure of carbon dioxide
PaO_2_: Arterial partial pressure of oxygen
PEEP: Positive end-expiratory pressure
pH: Potential of Hydrogen
PIP: Peak inspiratory pressure
RD: Respiratory distress
RDS: Respiratory distress syndrome
SAE: Serious adverse event
SAS: Silverman Andersen score
SpO_2_: Pulse oximetry oxygen saturation
TTN: Transient tachypnea of the newborn
